# PABPC1-induced stabilization of BDNF-AS inhibits malignant progression of glioblastoma cells through STAU1-mediated decay

**DOI:** 10.1038/s41419-020-2267-9

**Published:** 2020-02-03

**Authors:** Rui Su, Jun Ma, Jian Zheng, Xiaobai Liu, Yunhui Liu, Xuelei Ruan, Shuyuan Shen, Chunqing Yang, Di Wang, Heng Cai, Zhen Li, Yixue Xue

**Affiliations:** 1 Department of Neurobiology, College of Basic Medicine, China Medical University, Shenyang, 110122 China; 2 Key Laboratory of Cell Biology, Ministry of Public Health of China, China Medical University, Shenyang, 110122 China; 3 Key Laboratory of Medical Cell Biology, Ministry of Education of China, China Medical University, Shenyang, 110122 China; 4 Department of Neurosurgery, Shengjing Hospital of China Medical University, Shenyang, 110004 China; 5 Liaoning Clinical Medical Research Center in Nervous System Disease, Shenyang, 110004 China; 6 Key Laboratory of Neuro-oncology in Liaoning Province, Shenyang, 110004 China

**Keywords:** Targeted therapies, Long non-coding RNAs

## Abstract

Glioblastoma is the most common and malignant form of primary central nervous tumor in adults. Long noncoding RNAs (lncRNAs) have been reported to play a pivotal role in modulating gene expression and regulating human tumor’s malignant behaviors. In this study, we confirmed that lncRNA brain-derived neurotrophic factor antisense (BDNF-AS) was downregulated in glioblastoma tissues and cells, interacted and stabilized by polyadenylate-binding protein cytoplasmic 1 (PABPC1). Overexpression of BDNF-AS inhibited the proliferation, migration, and invasion, as well as induced the apoptosis of glioblastoma cells. In the in vivo study, PABPC1 overexpression combined with BDNF-AS overexpression produced the smallest tumor and the longest survival. Moreover, BDNF-AS could elicit retina and anterior neural fold homeobox 2 (RAX2) mRNA decay through STAU1-mediated decay (SMD), and thereby regulated the malignant behaviors glioblastoma cells. Knockdown of RAX2 produced tumor-suppressive function in glioblastoma cells and increased the expression of discs large homolog 5 (DLG5), leading to the activation of the Hippo pathway. In general, this study elucidated that the PABPC1-BDNF-AS-RAX2-DLG5 mechanism may contribute to the anticancer potential of glioma cells and may provide potential therapeutic targets for human glioma.

## Introduction

Glioma is the most common tumor type of the central nervous system with account for more than 50% of all primary brain tumors, and has been classified into I–IV grades of malignancy according to the World Health Organization (WHO)^1^. Glioblastoma is the most common and malignant type. The average survival of patients with glioblastoma is ~12–15 months due to the high invasion and proliferation^2^. Despite several efforts of surgical resection in combination with adjuvant chemoradiotherapy, patient prognosis remains poor. Currently, the field of glioblastoma research is focused on developing molecular targeted therapy that may provide new approaches for the treatment.

RNA-binding proteins have been identified as modulators of posttranscriptional mechanisms, including RNA splicing, transport, translation, and localization^3^. Numerous RBPs have been reported to be implicated in multiple tumor types, such as PCBP2 and Musashi 1 have been observed to contribute to glioma cells initiation and growth^4,5^. Polyadenylate-binding protein cytoplasmic 1 (PABPC1) is a cytoplasmic-nuclear shuttling protein expressed in most eukaryotes, which is important for protein translation initiation and decay. In addition, PABPC1 regulates mRNAs by binding to the poly(A) tails of them^6^. PABPC1 was corrected with tumor progression and patient's prognosis in esophageal cancer^7^. Besides, abnormal changes and functions of PABPC1 were found in cervical cancer (CC), colorectal cancer, and gastric cancer^8^. The expression and functional roles of PABPC1 in glioma remain unknown.

Long noncoding RNAs (LncRNAs) are defined as protein noncoding RNAs that, >200 nucleotides in length, have been shown to play important roles in various cancer development and progression. Multiple lncRNAs are identified as biomarkers for tumor diagnosis, treatment, or prognosis in recent years^9,10^. Brain-derived neurotrophic factor antisense (BDNF-AS) is a natural noncoding antisense of BDNF, locates at chromosome region 11p14.1, plays important roles in neuronal system together with BDNF^11^. Besides, BDNF-AS was lowly expressed in retinoblastoma (RB), non-small cell lung cancer (NSCLC) and CC, and suppressed cells proliferation and migration in these cancers^12^. The relationship between BDNF-AS and glioblastoma has not been found yet. The binding site between PABPC1 and BDNF-AS was predicted using the database of RBP specificities.

Stau1-mediated mRNA decay (SMD) is an upframeshift factor 1 (UPF1)-dependent mRNAs degradation mechanism, which is mediated by direct binding of STAU1 to STAU1-binding site (SBS) in mammalian cells^13^. STAU1 is a double-stranded RNA-binding protein, which can recognize SBSs in the 3′-UTR of target mRNAs and recruit UPF1 thereby mediated SMD^14^. SMD is a characteristic posttranscriptional regulation manner which has been reported to regulate a wide spectrum of physiological processes by degrading the expression of mRNAs involved. Cho et al. found that SMG1 can stimulate efficient adipogenesis by enhancing SMD^15^. In gastric cancer, lncRNA-TINCR caused KLF2 mRNA degradation via SMD thereby regulating gastric cancer cell proliferation and apoptosis^16^.

The retina and anterior neural fold homeobox 2 (RAX2) gene has 2428 bp in length and a cytogenetic location of 19p13.3, encodes 230 amino acids. As a member of transcription factor RAX, RAX2 is essential for eye development of several vertebrate species by modulating the proliferation and differentiation of retinal cells^17^. According to the prediction via software, an SBS were found between BDNF-AS and RAX2 mRNA 3'-UTR. Moreover, the JASPAR CORE database showed that RAX2 could bind to the promoter regions of discs large homolog 5 (DLG5). DLG5 belongs to the membrane associated guanylate kinase (MAGUK) family, participates in epithelial cell polarity maintenance and cancer development^18^. DLG5 expression was downregulated in many human cancers, such as bladder cancer, prostate cancer, breast cancer, and hepatocellular carcinoma, not only that, knockdown of DLG5 significantly increased cell migration and invasion in these cancers^19,20^.

In our study, we provided the endogenous expression and functions of PABPC1, BDNF-AS, RAX2, and DLG5 in human glioma tumor specimens and cells. Further, we explored their molecular mechanisms and effects on the biological behaviors of glioblastoma cells. The goal of this study was to demonstrate a novel molecular mechanism and experimental basis for the regulation of biological behaviors of glioblastoma cells, and provided promising therapeutic targets in human glioma.

## Materials and methods

### Cell culture

Normal human astrocyte (HA) cells were purchased from the ScienCell Research Laboratories (Carlsbad, CA, USA) and cultured in Roswell Park Memorial Institute (RPMI)–1640 supplemented with 10% fetal bovine serum (FBS, Gibco, Carlsbad, CA, USA). Human U87, U251 glioblastoma cell lines, and human embryonic kidney (HEK) 293T cells were purchased from Shanghai Institutes for Biological Sciences Cell Resource Center and cultured in high glucose Dulbecco’s modified Eagle’s medium supplemented with 10% FBS. All cells were maintained in a humidified incubator (5% CO_2_, at 37 °C).

### Human tissue samples

Glioma tissues and normal brain tissues (NBTs) were obtained from the patients who underwent surgery at the Department of Neurosurgery of Shengjing Hospital of China Medical University. Each patient was provided informed consent and the use of the samples for this study was approved by the Ethics Committee of Shengjing Hospital of China Medical University. All tumors were quickly frozen into liquid nitrogen at the time of resection and classified according to the WHO classification by neuropathologists.

### Cell transfections

The short-hairpin RNA directly against human BDNF-AS (NR_002832.2), PABPC1 (NM_002568.3), RAX2 (NM_001319074.1), or DLG5 (NM_004747.3) gene and their nontargeting sequences were ligated into pGPU6/GFP/Neo vectors (GenePharma, Shanghai, China), respectively. Human PABPC1 (NM_002568.3) mRNA sequence was ligated into the pcDNA3.1 vector. Human BDNF-AS gene and the negative control were ligated into pGCMV/MCS/IRES/EGFP/Neo vector (GenePharma, Shanghai, China). Full-length human DLG5 gene and respective nontargeting sequence were ligated into pIRES2-EGFP (GenScript, Piscataway, NJ, USA). The transfection of U87 and U251 cells was carried out using Lipofectamine 3000 and Opti-MEM (Life Technologies, Carlsbad, CA, USA). Geneticin (G418) and Puromycin (Sigma-Aldrich, St Louis, MO, USA) were used to select the stable-transfected cells.

### Western blotting

Western blot procedures were performed as previously described^21^. Proteins were detected by specific antibodies included PABPC1 (1:1000, Abcam, Cambridge, MA, USA), RAX2 (1:800, Proteintech Group, Rosement, USA), DLG5 (1:800, Proteintech Group, Rosement, USA), STAU1 (1:1000, Proteintech Group, Rosement, USA), UPF1 (1:1000, Proteintech Group, Rosement, USA), GAPDH (1:5000, Cell Signaling Technology, Danvers, MA, USA), or Hippo Signaling Antibody Sampler Kit (Cell Signaling Technology, Danvers, MA, USA).

### RNA extraction and quantitative real-time PCR (qRT-PCR)

Total RNA was extracted using the Trizol reagent (Life Technologies Corporation, Carlsbad, CA, USA), and quantified using a NanoDrop^TM^ 3000 Spectrophotometer (Thermo Fisher Scientific, USA). QRT-PCR was conducted using One-Step SYBR PrimerScript RT-PCR Kit (TakataBio, Inc., Japan) according to the manufacturer’s protocol. See Additional file 1 for details and primers used.

### LncRNAs microarray

LncRNAs analysis and microarray hybridization were performed by Kangchen Bio-tech (Shanghai, China).

### Nascent RNA capture assay

To capture the newly synthesized RNA transcripts of BDNF-AS and RAX2 mRNA, the nascent RNA capture assay was carried out using the Click-iT Nascent RNA Capture Kit (Thermo Fisher Science, Waltham, MA, USA) according to the manufacturer’s protocol. Briefly, cells were seeded at 40–50% confluency and incubated with appropriate volume of 5-ethynyluridine (EU) solution at 37 °C at 5% CO_2_ for 24 h. Cells were harvest and then centrifuged and total RNA was isolated as described before. Dynabeads^®^ MyOne™ Streptavidin T1 magnetic beads were used in biotinylated total RNA to pull out the biotin-labeled EU-RNA. After removing the supernatant and washing the beads using wash buffer, the nascent RNA captured on the beads was analyzed using qRT-PCR as described.

### RNA stability assay

To measure the stability of BDNF-AS, cells were treated with 5 μg/ml actinomycin D (Sigma, MO, USA) which could block transcription. Cells were collected at 0–5 h after addition of actinomycin D, and the total cellular RNA was isolated. QRT-PCR was performed to measure the half-life of BDNF-AS and GAPDH mRNA was applied as an internal control.

### RNA-protein immunoprecipitation (RIP) assay

RIP were carried out using the EZ-Magna RIP Kit (Millipore, Billerica, MA, USA) according to the manufacturer’s instruction. Cells were lysed by a complete RNA lysis buffer with protease inhibitor and RNase, and then incubated with RIP buffer containing magnetice beads conjugated with antibodies of STAU1 with negative control (IgG), respectively. Wash buffer was added to all RIP reactions and vortex gently, and repeat wash steps until magnetic beads have been washed a total of five times. The fold enrichments of immunoprecipitated RNAs with different antibodies were analyzed using qRT-PCRs when the immunoprecipitated RNA was isolated and purified.

### Cell proliferation assay

Cell proliferation was examined with the Cell Counting Kit-8 (CCK-8) assay using a Cell Counting Kit (Beyotime, Jiangsu, China) as previously described^21^.

### Apoptosis assay

Apoptosis rate was routinely determined by flow cytometry using Annexin V-PE/PI staining (Southern Biotech, Birmingham, AL). Briefly, cells were harvested and stained with Annexin V-FITC and PI according to the manufacturer’s instruction. Data analysis was performed using BD accuri C6 software.

### Migration and invasion assay

The migration and invasion assays were carried out using transwell chambers (Corning, NY, USA, 3422, 8-μm pores) according to previously described^21^.

### RNA pull-down assay

Biotin-labeled, full-length BDNF-AS RNA, or BDNF-AS with mutational SBS (BDNF-AS-SBS-Mut) fragment was prepared with the Biotin RNA Labeling Mix (GenePharma, Shanghai, China) and transfected into U87 and U251 cells. Biotinylated RNAs were treated with RNase-free DNase I and purified. RNA-protein complexes were isolated by streptavidin agarose beads (Invitrogen, Shanghai, China) and washed three times. The retrieved proteins were detected using a standard western blotting technique as described above.

### Reporter vectors constructs and luciferase assay

BDNF-AS full-length and RAX2 3'-UTR sequences were amplified by PCR and cloned in the pmirGlo Dual-luciferase Vector (Promega, Madison, USA) to construct luciferase reporter vector (BDNF-AS-Wt and RAX2-Wt) (GenePharma, Shanghai, China). The sequence of putative binding site of BDNF-AS (ALU: 1982–2062bp) and RAX2 (ALU: 2116–2419 bp) was replaced as indicated (BDNF-AS-Mut, RAX2-Mut). The responsive RAX2-binding sites in the DLG5 promotor were predicted by bioinformatics tool JASPAR and were determined by dual-luciferase reporter system. Promoter fragments were subcloned into pGL3-Basic-Luciferase vector (Promega, WI, USA). Human full-length RAX2 was constructed in pEX3 vector (GenePharma, Shanghai, China). The assay was performed 48 h after transfection the indicated constructs into 2.4 × 10^4^ HEK-293T cells per well seeded into 96-well plates. Cells were analyzed by the luciferase assay using the dual-luciferase reporter assay system. The relative luciferase activity was expressed as the ratio of firefly luciferase activity to renilla luciferase activity.

### Nude mouse xenograft model

For the in vivo study, cells stably transfected with PABPC1(+), BDNF-AS(+), PABPC1(+) + BDNF-AS(+) were selected. Four weeks old female BALB/C nude mice were purchased from Cancer Institute of the Chinese Academy of Medical Science and divided into five groups by chance (*n* = 6, each group) for subcutaneous and intracranial orthotropic inoculation, respectively. A total of 3 × 10^5^ cells were subcutaneously inoculated in the left flank of the mice. Tumor nodules were estimated every 5 days after injection. The following formula was used to calculated tumor volume: volume (mm^3^) = length × width^2^/2. For intracranial orthotropic inoculation, 3 × 10^5^ cells were injected into the right striatum of nude mice. Then, the number of survived nude mice was recorded every 5 days. Survival analysis was carried out in the form of Kaplan–Meier survival curve. All animal experiments were complied with the Guide for the Care Committee of the Shengjing Hospital.

### Chromatin immunoprecipitation (ChIP) assay

Chromatin was fixed and immunoprecipitated using the Simple ChIP Enzymatic Chromatin IP Kit (Cell signaling Technology, MA, USA) as recommended by the manufacturer. See Additional file 1 for details and primers used.

### Statistical analysis

In this work, all data were described as mean ± SD from at least three times independent experiments. All statistical analysis was conducted using SPSS 18.0 statistical software with student’s *t* test (between two groups) or one-way ANOVA analysis (three or more groups) of variance. Differences were considered as statically significant when *P* < 0.05.

## Results

### PABPC1 acted as a tumor suppressor in glioblastoma cell lines

**Fig. 1:**
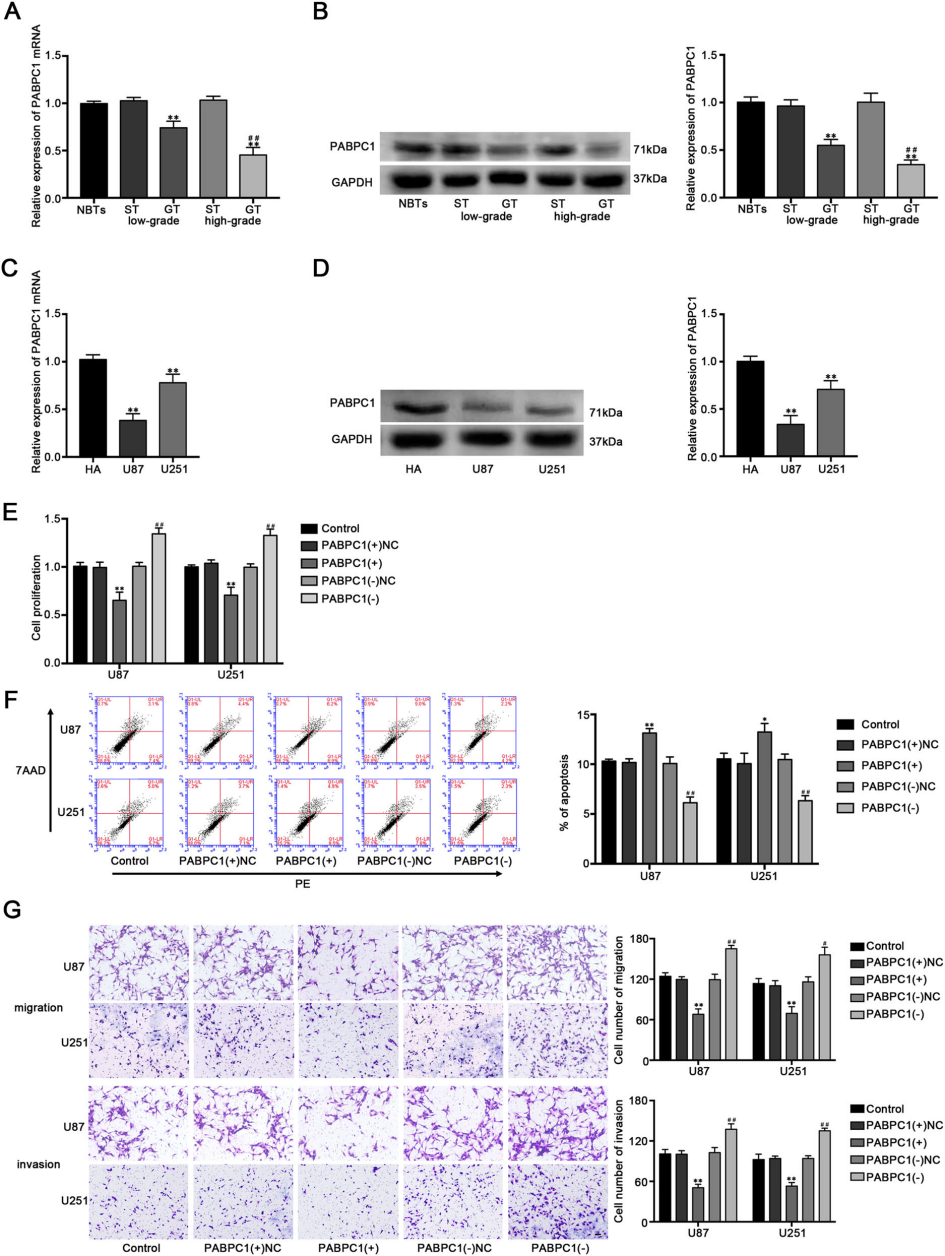
The expression and effects of PABPC1 in glioblastoma cells. **a** The PABPC1 mRNA expression levels in normal brain tissues (NBTs), low and high grades of human glioma tissues (GT), and homologous surrounding nonneoplastic tissues (ST). **b** The PABPC1 protein expression levels in NBTs, low and high grades of GT and homologous ST (*n* = 4, each group). **P < 0.01 vs. ST group; ^##^*P* < 0.01 vs. low-grade GT group. **c** The mRNA expression level of PABPC1 in human astrocytes (HA) and glioblastoma cell lines (U87 and U251). **d** The protein expression level of PABPC1 in human astrocytes (HA) and glioblastoma cell lines (U87 and U251). (*n* = 3, each group). **P < 0.01 vs. HA group. **e** The CCK-8 assay was used to measure the effect of PABPC1 on the proliferation of U87 and U251 cells. **f** The apoptotic percentages of U87 and U251 cells were detected after PABPC1 overexpression or knockdown. **g** The transwell assays were used to measure the effect of PABPC1 on cell migration and invasion of U87 and U251 cells. Scale bars represent 40 μm. (*n* = 5, each group). *P < 0.05 or **P < 0.01 vs. PABPC1(+) NC group; ^#^*P* < 0.05 or ^##^*P* < 0.01 vs. PABPC1(−)NC group.

By using the Oncomine database (https://www.oncomine.org/resource/main.html), the lower expression of PABPC1 in glioblastoma tissues compared with neural stem cells were found (Fig. S1A). We further examined the expression levels of PABPC1 in human glioma tissues (GT) and cell lines by qRT-PCR and western blot. As shown in Fig. 1a–d, PABPC1 expressed lower in GT and cells than in surrounding nonneoplastic tissues (ST) and NBTs, and the expression level was negatively correlated with the histopathological grades of gliomas. Furthermore, PABPC1 expression was significantly lower in U87 and U251 cells than in HA cells. Stable PABPC1 overexpressed and silenced constructs were used to further evaluate the biological role (Fig. S1B). As shown in Fig. 1e, the proliferation ability of glioblastoma cells was decreased in the PABPC1(+) group, while increased in the PBAPC1(−) group compared with their nonspecific control (NC) group, respectively. Overexpression of PABPC1 significantly increased the apoptosis ratio of glioblastoma cells (Fig. 1f) and inhibited the migration and invasion capability in glioblastoma cells (Fig. 1g). These data suggested that PABPC1 functioned as a tumor suppressor in glioblastoma cells.

### Overexpression of BDNF-AS inhibited malignant behaviors of glioblastoma cells

**Fig. 2:**
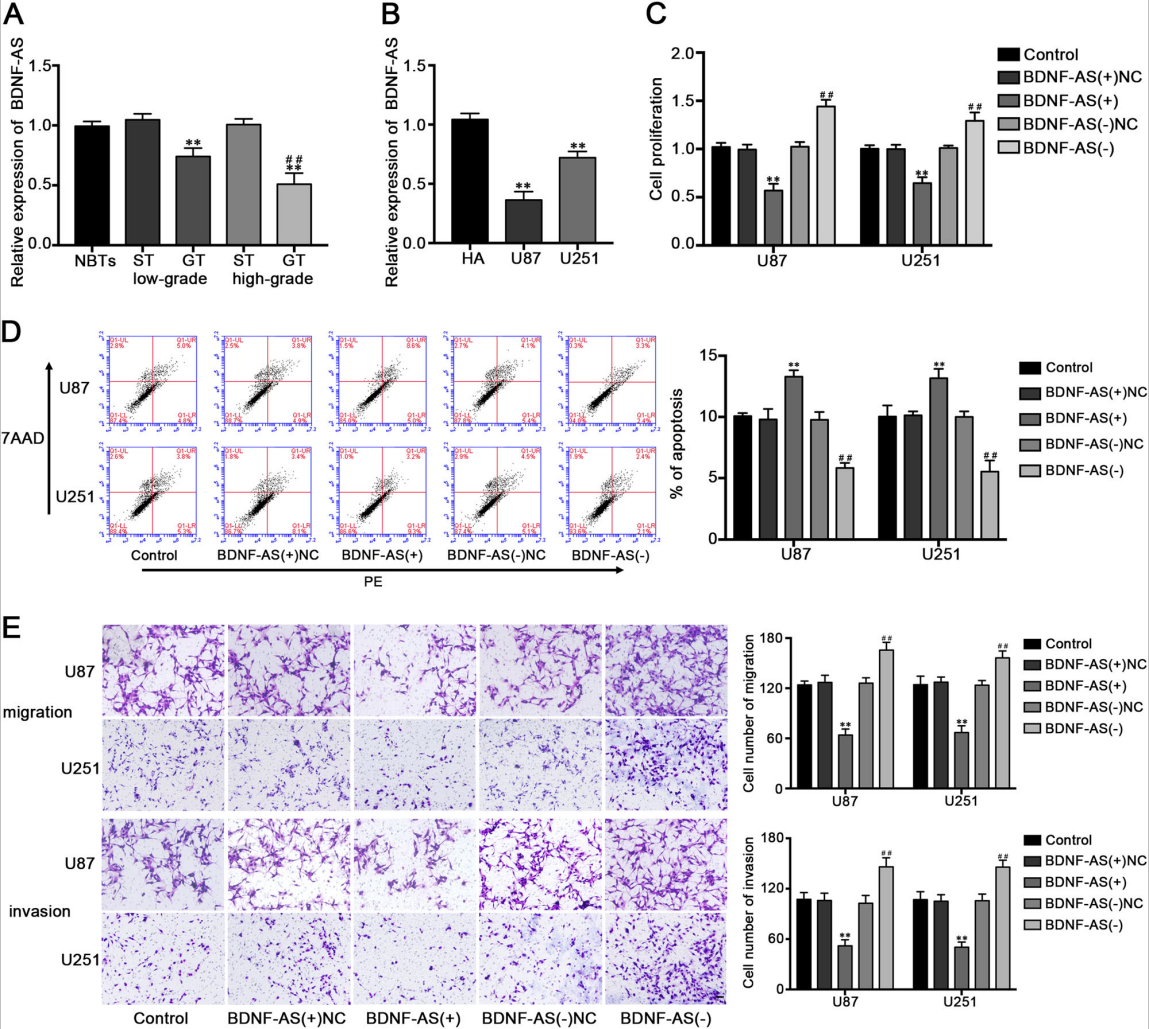
The expression and effects of BDNF-AS in glioblastoma cells. **a** The relative expression levels of BDNF-AS in NBTs, low and high grades of human glioma tissues. Data are presented as the mean ± SD (*n* = 4, each group). **P < 0.01 vs. ST group; ^##^*P* < 0.01 vs. low-grade GT group. **b** The relative expression levels of BDNF-AS in HA and glioblastoma cell lines. Data are presented as the mean ± SD (*n* = 3, each group). **P < 0.01 vs. HA group. **c** Effect of BDNF-AS on the cell proliferation, cell apoptosis (**d**), cell migration and invasion (**e**) of glioblastoma cells. Scale bars represent 40 μm. (*n* = 5, each group). **P < 0.01 vs. BDNF-AS(+)NC group; ^##^*P* < 0.01 vs. BDNF-AS(−)NC group.

QRT-PCR was performed to evaluate BDNF-AS expression levels in GT and cells, and the results indicated that BDNF-AS was downregulated in GT and cell lines compared with NBTs and HA cells, respectively. Moreover, the expression level of BDNF-AS in GT was negatively correlated with histopathological grade in human GT (Fig. 2a, b). To determine the effects of BDNF-AS on glioblastoma cells, the stable overexpression and knockdown of BDNF-AS of U87 and U251 cell lines were established, the transfection efficiency were shown in Fig. S1C. The CCK-8 assay manifested that the overexpression of BDNF-AS inhibited the proliferation of U87 and U251 cells (Fig. 2c). Flow cytometry analysis results showed that the apoptosis of U87 and U251 cells was increased in BDNF-AS(+) group compared with the BDNF-AS(+)NC group (Fig. 2d). Moreover, as showed in Fig. 2e, BDNF-AS overexpression significantly inhibited the migration and invasion capabilities in glioblastoma cells. In the meantime, knockdown of BDNF-AS exerted opposite effects in same assays. We proposed that BDNF-AS exerted tumor-suppressive function in glioblastoma cells.

### PABPC1 increased the stability of BDNF-AS and interacted with BDNF-AS in glioblastoma cells

**Fig. 3:**
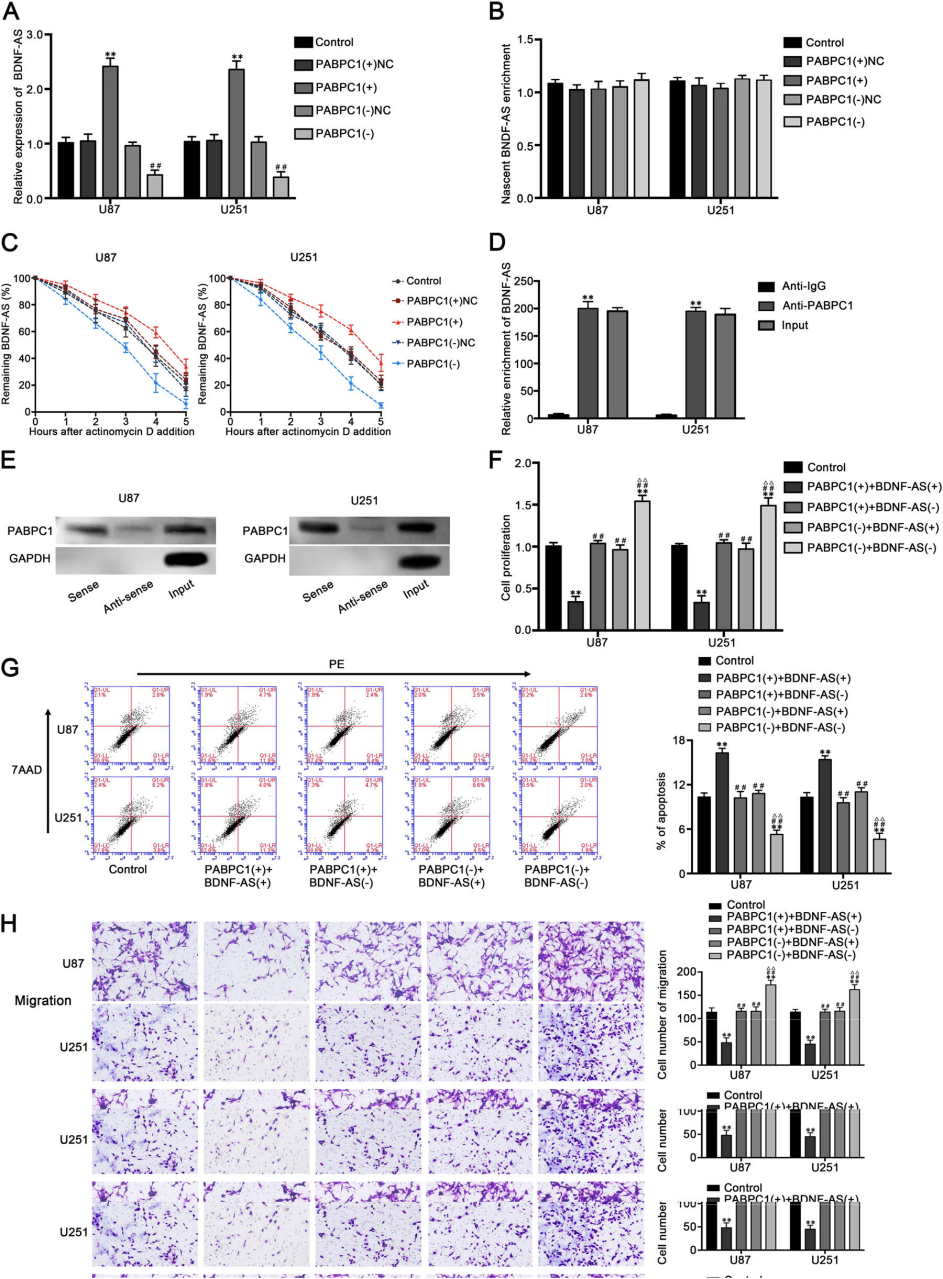
PABPC1 binds to BDNF-AS and stabilizes BDNF-AS. **a** Expression of BDNF-AS after PABPC1 overexpression or knockdown (*n* = 3, each group). **P < 0.01 vs. PABPC1(+)NC group. ^##^*P* < 0.01 vs. PABPC1(−)NC group. **b** Identification of the newly synthesized RNA enrichment of BDNF-AS via RNA capture assay and qRT-PCR. **c** Half-life of BDNF-AS measured by qRT-PCR and normalized to the level of GAPDH. **d** RIP was performed in U87 and U251 cells and followed by qRT-PCR to detect BDNF-AS associated with PABPC1. **P < 0.01 vs. anti-IgG group. **e** Western blot analysis following RNA pull-down assays performed using U87 and U251 cellular exacts. **f** Effect of PABPC1 and BDNF-AS on cell proliferation, **g** apoptosis, **h** migration and invasion of glioblastoma cells. Scale bars represent 40 μm. (*n* = 5, each group). **P < 0.01 vs. control group; ^##^*P* < 0.01 vs. PABPC1(+) + BDNF-AS(+) group. ^▵▵^*P* < 0.01 vs. PABPC1(+) + BDNF-AS(−) group.

Using the RNA-binding protein database and lncRNAs microarray analysis, we found the putative binding between PABPC1 and many lncRNAs, and we found that BDNF-AS was significantly upregulated by the overexpression of PABPC1 (Fig. S1D). Therefore, we proposed that BDNF-AS might be involved in the regulation of PABPC1 on glioma cells. Our results showed that the expression of BDNF-AS was increased in PABPC1(+) group, whereas decreased in PABPC1(−) group (Fig. 3a). Since PABPC1 was confirmed to increase the stability of mRNAs or LncRNAs^22^, we hypothesized that PABPC1 increases the expression of BDNF-AS by increasing the stability of it. As shown in Fig. 3b, c, nascent RNA capture assays showed no significant effect of PABPC1 on BDNF-AS RNA synthesis, whereas PABPC1 overexpressing significantly increased the half-life of BDNF-AS. These results indicated that PABPC1 regulated BDNF-AS expression at a posttranscriptional level. To confirm whether PABPC1 interacts with BDNF-AS in U87 and U251 cells, RNA immunoprecipitation (RIP) and RNA pull-down assays were carried out. As show in Fig. 3d, RIP was performed with an antibody against PABPC1 using extracts from glioblastoma cells and BDNF-AS enrichment was observed to increase in anti-PABPC1 group than in anti-IgG group significantly. Then, western blot analysis using proteins form RNA pull-down assays confirmed that PABPC1 is associated with BDNF-AS compared with antisense group (Fig. 3e). Moreover, the stable BDNF-AS overexpressed and silenced constructs were transfected into PABPC1(+) group and PABPC1(−) group cells respectively to investigate the role of BDNF-AS on the function of PABPC1. As shown in Fig. 3f–h, silencing of BDNF-AS counteract the effect of the overexpression of PABPC1 on cell proliferation, migration, invasion, and apoptosis, implied that BDNF-AS is associated with the PABPC1-induced effects on glioblastoma cells.

### The combination of PABPC1 overexpression and BDNF-AS overexpression suppressed tumor growth and exhibited high survival time in nude mice

**Fig. 4:**
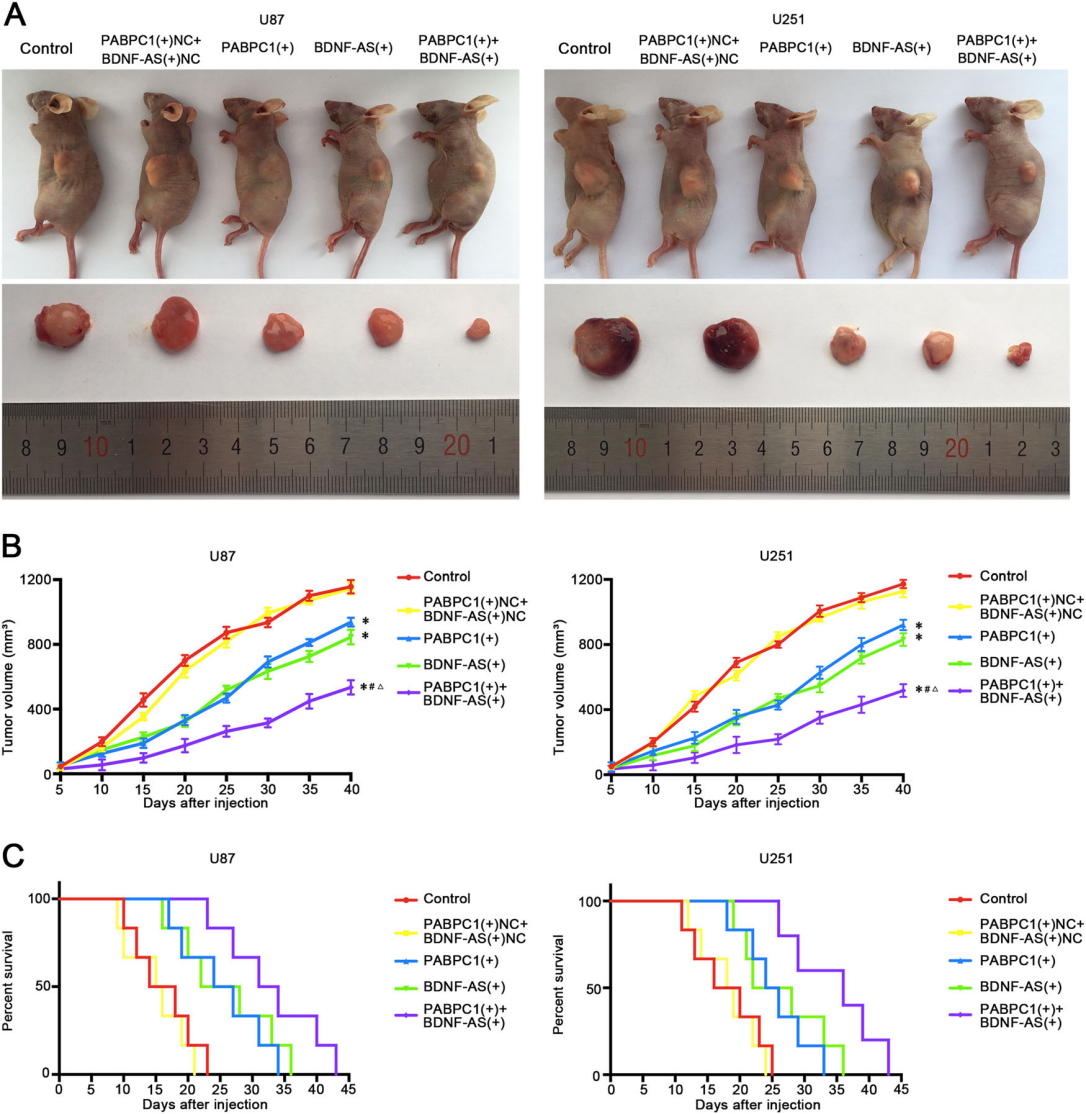
Tumor xenograft experiments. **a** The nude mice carrying tumors from respective groups were shown and the sample tumors from respective group were shown. **b** Tumor growth curves were shown. Tumor volume was calculated every 5 days after injection, and the tumor was taken after 40 days. **c** Survival curves from respective nude mice injected into the right striatum were shown (*n* = 6, each group).

To further explore the antitumor function of PABPC1 and BDNF-AS in vivo, the stable expression cells were used in nude mice. As shown in Fig. 4a, b, PABPC1 overexpression, BDNF-AS overexpression, and combination of them produced lower tumors than the control group. In addition, PABPC1 overexpression combined with BDNF-AS overexpression led to the smallest tumor among all groups. Similarly, survival analysis indicated that mice in PABPC1(+) + BDNF(+) group had the longest survival time (Fig. 4c).

### Knockdown of RAX2 inhibited the cell proliferation, migration and invasion, and promoted apoptosis of glioblastoma cells

**Fig. 5:**
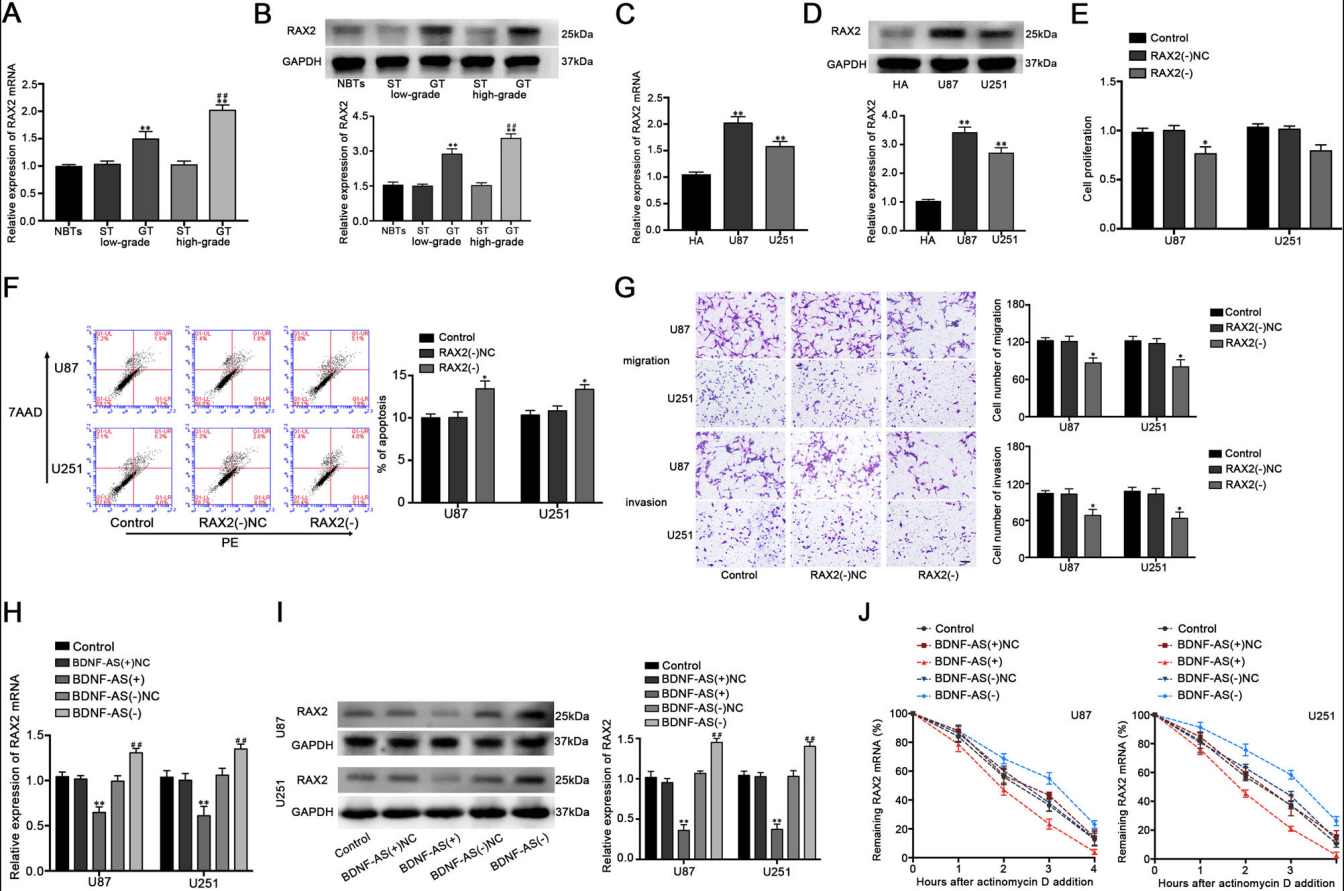
The expression and effects of RAX2 in glioblastoma cells. **a** The mRNA and **b** protein expression level of RAX2 in NBTs, low and high grades of GT and homologous ST (*n* = 4, each group). **P < 0.01 vs. ST group; ^##^*P* < 0.01 vs. low-grade GT group. **c** The mRNA and **d** protein expression level of RAX2 in HA, U87, and U251 (*n* = 3, each group). **P < 0.01 vs. HA group. **e** Effect of RAX2 knockdown on the cell proliferation of U87 and U251 cells. **f** Effect of RAX2 knockdown on the cell apoptosis of U87 and U251 cells. **g** Effect of RAX2 knockdown on the cell migration and invasion of U87 and U251 cells. Scale bars represent 40 μm. (*n* = 5, each group). *P < 0.05 vs. RAX2(−)NC group. **h** Expression levels of RAX2 mRNA and **I** protein after BDNF-AS overexpression or knockdown (*n* = 3, each group). **P < 0.01 vs. BDNF-AS(+)NC group. ^##^*P* < 0.01 vs. BDNF-AS(−)NC group. **j** The half-life of RAX2 mRNA were estimated after BDNF-AS overexpression or knockdown (*n* = 3, each group).

QRT-PCR and western blot were used to analyze the expression level of RAX2 in human GT and cells. The results showed that RAX2 was upregulated in GT and cell lines and the expression level in tissues was positively correlated with histopathological grade of GT (Fig. 5a–d). This increase of RAX2 expression prompted that RAX2 may have possible biological effects on glioblastoma cells, therefore the biological behaviors of U87 and U251 cells were detected after stably constructing RAX2 silenced cells (Fig. S1E). As shown in Fig. 5e–g, RAX2 knockdown inhibited cell proliferation, migration and invasion, and promoted the cell apoptosis of U87 and U251 cells. These results indicated that knockdown of RAX2 exerts tumor-suppressive effects in human glioblastoma cells.

### BDNF-AS elicited RAX2 mRNA decay through SMD

**Fig. 6:**
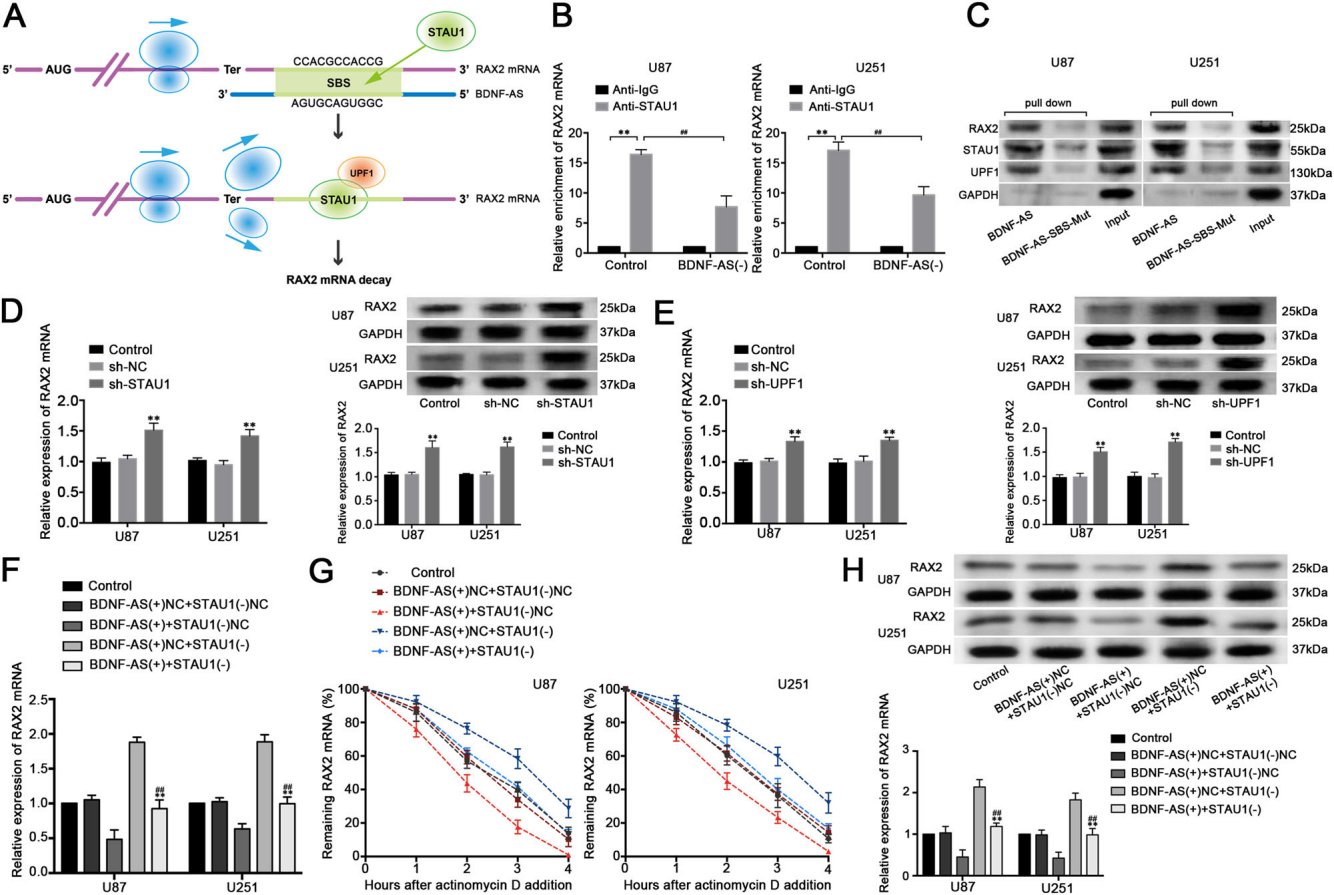
BDNF-AS affects RAX2 mRNA expression and stability through SMD. **a** Diagrams of the mechanism of SMD. A predicted imperfect base pairing (green) between Alu elements of RAX2 mRNA (purple) and BDNF-AS forms SBS. STAU1 (green ellipse) can recognize SBS and recruit UPF1 (orange ellipse) to trigger SMD and cause degradation of RAX2 mRNA. **b** Effects of BDNF-AS knockdown on the relative enrichment of RAX2 mRNA in IgG or STAU1 precipitates (*n* = 3, each group). **P < 0.01 vs. Anti-IgG control group. ^##^*P* < 0.01 vs. Anti-STAU1 control group. **c** U87 and U251 cells were transfected with biotinylated BDNF-AS (BDNF-AS) and BDNF-AS with mutational SBS (BDNF-AS-SBS-Mut), and western blot analysis following RNA pull-down assays performed using cellular exacts. **d** Effect of STAU1 and **e** UPF1 on the mRNA and protein expression of RAX2 in U87 and U251 cells (*n* = 3, each group). **P < 0.01 vs. sh-NC group. **f** Effects of the combination of overexpressed BDNF-AS and the decreased STAU1 on mRNA expression level, **g** stability and **h** protein expression level (*n* = 5, each group). **P < 0.01 vs. BDNF-AS(+)+STAU1(−)NC group. ^##^*P* < 0.01 vs. BDNF-AS(+)NC+STAU1(−) group.

Several studies indicated that lncRNAs are functionally in regulating many fundamental cellular process including affecting mRNA expression^23^. We revealed that the overexpression of BDNF-AS decreased RAX2 expression and mRNA half-life, while the knockdown of BDNF-AS produced opposite effects (Fig. 5h–j). Moreover, we captured the nascent RAX2 mRNA and found no significant changes among each group (Fig. S2A). Recent studies reported that STAU1 could bind to the intermolecular base pairing in the 3'-UTR of the target mRNA and cause a decay at posttranscriptional level^24^. Interestingly, two Alu elements on BDNF-AS and 3'-UTR of RAX2 mRNA were found using bioinformatic software Repeatmasker (http://www.repeatmasker.org/cgi-bin/WEBRepeatMasker) respectively, and a putative imperfect base pairing between them was predicted by IntaRNA (http://rna.informatik.uni-freiburg.de/IntaRNA/Input.jsp, Fig. 2b). This made us speculated that whether BDNF-AS caused RAX2 mRNA degradation through SMD. RIP assays with IgG and STAU1 antibodies were used to investigate the association between BDNF-AS and RAX2 mRNA. As shown in Fig. S2C, BDNF-AS and RAX2 mRNA were enriched in anti-STAU1 groups compared with the anti-IgG control groups, proving exist of the binding between STAU1 with BDNF-AS and RAX2 mRNA, respectively. Moreover, knockdown of BDNF-AS decreased the association between STAU1 and RAX2 mRNA, suggesting that BDNF-AS is wanted for the combination between STAU1 with RAX2 mRNA (Fig. 6b). Cells were further transfected with biotinylated BDNF-AS and BDNF-AS with mutational SBS variants and extracted for pull-down assays. As expected, RAX2, STAU1, and UPF1 were enriched in BDNF-AS groups, while few were observed in BDNF-AS-SBS-Mut groups (Fig. 6c). This indicated that the relation among BDNF-AS, RAX2, and STAU1 is based on the predicted SBS sequence in BDNF-AS, and the SMD factor UPF1 is participated. Furthermore, as shown in Fig. 6d, e, loss of STAU1 and UPF1 increased RAX2 expression in both mRNA and protein levels. Consistently, knockdown of STAU1 restore the inhibiting effects of BDNF-AS on RAX2 mRNA expression and half-life time, including RAX2 protein expression (Fig. 6f–h). These results suggested that BDNF-AS affects RAX2 mRNA expression through SMD.

### DLG5 was downregulated in glioma tissues and cell lines, and functioned as tumor suppressor in glioblastoma cells

**Fig. 7:**
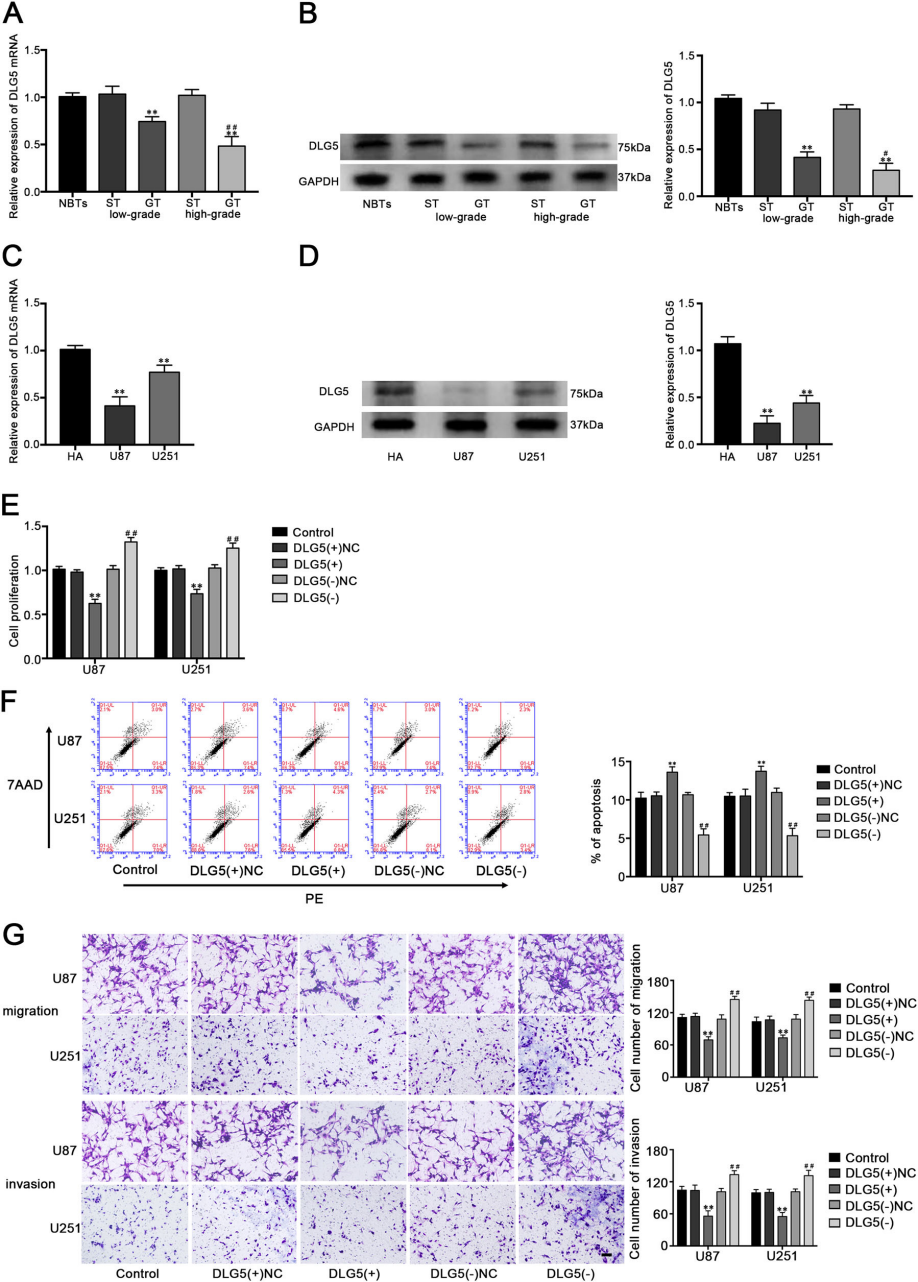
Expression of DLG5 in glioma tissues and cell lines and the effects of DLG5 on glioblastoma cells. **a** The mRNA and **b** protein expression level of DLG5 in normal and glioma tissues (n = 4, each group). **P < 0.01 vs. ST group; ^##^*P* < 0.01 vs. low-grade GT group. **c** The mRNA and **d** protein expression level of DLG5 in HA, U87 and U251 (n = 3, each group). **P < 0.01 vs. HA group. **e** Effect of DLG5 on the cell proliferation, **f** cell apoptosis, **g** cell migration and invasion of glioblastoma cells (n = 5, each group). **P < 0.01 vs. DLG5(+)NC group; ^##^*P* < 0.01 vs. DLG5(−)NC group.

QRT-PCR and western blot assays were used to measure the expression level of DLG5 and it was significantly decreased in GT and cell lines, and more downregulated in high-grade GT compared with low-grade GT (Fig. 7a–d). To further explore the impact of DLG5 on glioblastoma cell lines, a series of assays were carried out after DLG5 overexpression or knockdown. CCK-8 assay indicated that overexpression of DLG5 inhibited the proliferation of U87 and U251 cells, whereas DLG5 knockdown exerted the opposite effect (Fig. 7e). Cell apoptosis in the DLG5(+) group was promoted, and inhibited in the DLG5(−) group compared with their respective NC group (Fig. 7f). Transwell assays showed that the migration and invasion U87 and U251 cell numbers were apparently decreased in DLG5(+) group and increased in DLG5(−) group than in respective NC group (Fig. 7g). We proposed that DLG5 may exert tumor-suppressive function in glioblastoma cells.

### RAX2 suppressed DLG5 expression by directly binding to its promoter

**Fig. 8:**
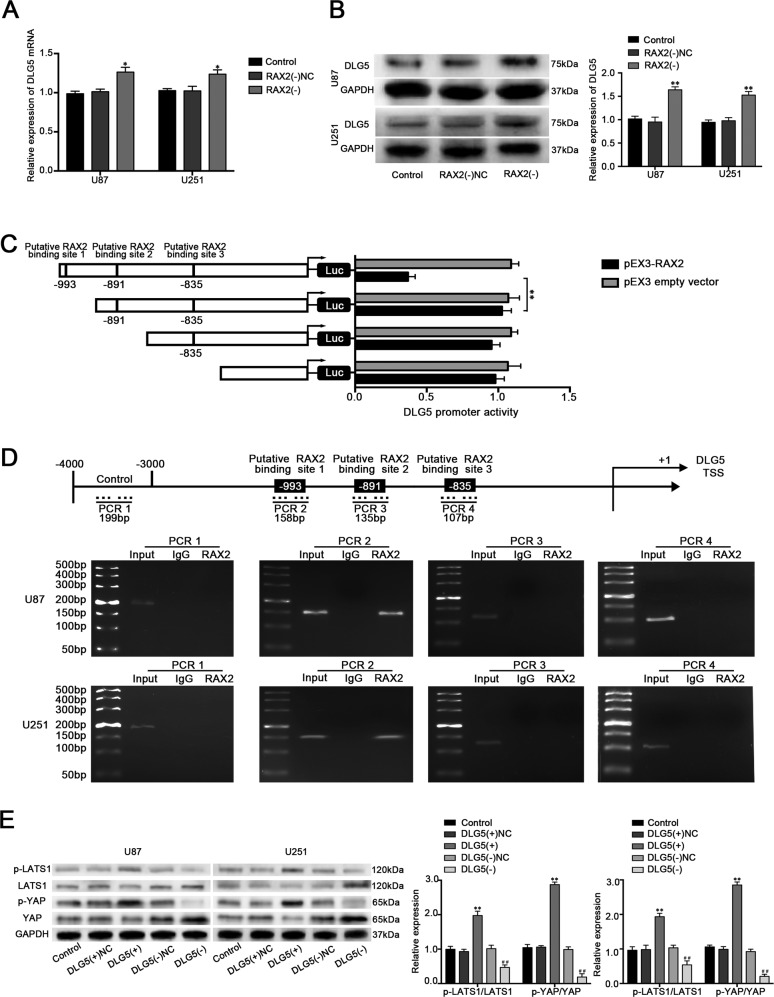
RAX2 increased the expression of DLG5 by binding to the promoter of DLG5. **a** The DLG5 mRNA expression after RAX2 knockdown was detected via qRT-PCR. **b** Western blot assay was used to detect the protein expression of DLG5 after RAX2 knockdown (*n* = 3, each group). **P < 0.01 vs. RAX2(−)NC group. **c** RAX2 affect on DLG5 promoter activity. **d** RAX2 bound to the promoter of DLG5 in U87 and U251 cells. Schematic representation of the human DLG5 promoter region 4000 bp upstream of the transcription start site (TSS), which was designated as +1. Putative RAX2-binding site was indicated. PCR was conducted with the resulting precipitated DNA. **e** Western blot assay of the p-LATS1/LATS and p-YAP/YAP expression regulated by DLG5. **P < 0.01 vs. DLG5(+)NC group; ^##^*P* < 0.01 vs. DLG5(−)NC group.

Our previous results showed that RAX exerted an oncogenic factor in glioblastoma cells. Additionally, RAX2 as a transcription factor may transcriptionally regulated the downstream gene in controlling behaviors of glioblastoma cells. DLG5 promoter was predicted that it has binding sites of RAX2 by bioinformatic database JASPAR (Fig. S2D). As shown in Fig. 8a–b, DLG5 expression was increased in the RAX2(−) group compared with RAX2(−)NC group. By analyzing the DNA sequence of DLG5 promoter in the region from 1000 bp upstream to 100 bp downstream of transcription start site, three putative binding sites of RAX2 were found. As shown in Fig. 8c, deletion of the putative binding site 1 (−993 bp site region) significantly upregulated the promoter activities of DLG5. ChIP assay was performed subsequently and the results showed that there was an interaction of RAX2 with putative binding site 1 of DLG5 and there was no association of RAX2 with the control region (Fig. 8d). DLG5 was identified to have connection with the Hippo pathway^25^. In addition, loss of DLG5 promoted cancer malignancy by inactivating the Hippo pathway in breast cancer^26^. To elucidate whether DLG5 could suppress glioblastoma progression by affecting Hippo pathway, the activity of Hippo pathway was detected. As shown in Fig. 8e, p-LATS1 and p-YAP expression was increased in DLG5(+) group while reduced in DLG5(−) group. These results indicated that DLG5 could up-regulate the activity of the Hippo pathway in glioblastoma cells.

## Discussion

In this study, we identified PABPC1 and BDNF-AS the significantly downregulated expression in GT and cells. Overexpression of PABPC1 and BDNF-AS inhibited the cell proliferation, migration and invasion, and promoted apoptosis of glioblastoma cells. PABPC1 interacted with BDNF-AS and increased the expression of BDNF-AS by stabilizing it. BDNF-AS bound to the 3'UTR of RAX2 mRNA and reduced RAX2 mRNA through SMD. RAX2 expression was elevated in glioma and knockdown RAX2 inhibited the malignant biological behaviors of glioblastoma cells. Moreover, RAX2 could increase DLG5 expression through the level of transcription. Overexpression of DLG5 inhibited glioblastoma tumor malignancy by activating the Hippo signaling pathway. We demonstrated for the first time that the PABPC1/BDNF-AS/RAX2/DLG5 pathway plays the vital role in regulating the malignant biological behaviors of glioblastoma cells.

PABPC1 is a classical complex cytoplasmic polyadenylate-binding protein involved in mRNA biogenesis, mRNA turnover and mRNA deadenylation. In addition, PABPC1 plays important roles in promoting protein translation initiation and ribosome recruitment^27^. In eukaryotic cytoplasm, PABPC1 stabilizes RNA by covering the 3' poly (A) tail of mRNA through RNA recognition motifs and interacting with eukaryotic initiation factor 4G^28^. PABPC1 is located at chromosome region 8q22.3, and the loss of heterozygosity at this region is associated with occurrence of oral cancer and breast cancer^29,30^. The abnormally expression of PABPC1 was found in many human malignancies, such as esophageal squamous cell carcinoma (ESCC), CC, colorectal cancer, and gastric cancer^8,31,32^. In this study, we found that PABPC1 was downregulated in human GT and cell lines, especially in high grade. Overexpression of PABPC1 inhibited cell proliferation, migration, and invasion, while promoted apoptosis in glioblastoma cells; knockdown PABPC1 produced the opposite effect. These results suggested that PABPC1 acted as a tumor suppressor in glioblastoma cells. Similarly, the decreased expression of PABPC1 was found in ESCC, which is correlated with poorer prognosis of patients and local invasiveness in tumor tissues^7^.

Increasing evidence has shown lncRNAs can act as either tumor oncogenes or suppressors by regulating tumor progression including glioma^33,34^. BDNF-AS is a naturally conserved lncRNA that inhibits the expression of BDNF by decreasing BDNF mRNA levels and functions opposite to BDNF^35,36^. BDNF has been reported to be upregulated and act as a predominant oncogenic factor in a variety human cancers^37,38^. Interestingly, BDNF-AS was demonstrated to be reversely associated with BDNF and downregulated expressed in RB, NSCLC, and CC^12,39,40^. Xiong et al. found that mature BDNF induces glioma cells in vitro^41^. However, little is known about the expression or functions of BDNF-AS in human glioma. We evaluated that BDNF-AS was significantly downregulated in GT and cell lines, and found that BDNF-AS overexpression resulted in the inhibition of malignant biological behaviors of glioblastoma cells; knockdown BDNF-AS had the opposite effect. Similarly, overexpression of BDNF-AS induced cell cycle arrest at G0/G1 phase and inhibited cell proliferation and migration in RB^39^. Moreover, BDNF-AS inhibited cell viability by forced overexpression in NSCLC and CC^12,40^.

Since PABPC1 and BDNF-AS were both downregulated and act as tumor suppressors in glioblastoma cells, we wondered whether PABPC1 would exert direct action on BDNF-AS. Based on predictions of bioinformatics database, we firstly demonstrated that PABPC1 could bind to BDNF-AS and increased the expression of BDNF-AS by stabilizing it at a posttranscriptional level. Moreover, we revealed that tumor-suppressive effects of PABPC1 were mediated by BDNF-AS in glioblastoma cell lines by a series of assays. PABPC1 is known to be involved in regulating stabilities of mRNA and also lncRNAs^42^. For example, PABPC1 stabilized and increased the half-life of lncRNA-PAN by interacting with lncRNA-PAN through forming a complex with the Kaposi’s sarcoma-associated herpesvirus ORF57 protein beforehand^43^. In gallbladder cancer (GBC), PABPC1 was demonstrated to regulate tumorigenesis in GBC cells by interacting and stabilizing lncRNA-PAGBC^22^.

Furthermore, the in vivo studies also demonstrated that PABPC1 overexpression, BDNF-AS overexpression, and the combination of the above observably suppressed tumor growth and prolonged the survival days in nude mice. Among them, the mice carrying the combined treatment produced the significantly smallest tumors and had the highest survival, suggesting that the combination of PABPC1 overexpression and BDNF-AS overexpression could be potentially applied in the treatment of glioma.

Rax is a novel homeobox gene which controls development of the eye and forebrain^44^. Rax encoded transcription factor RAX is essential for vertebrate eye development, which also has been shown to act with cone-rod homeobox to control gene expression in retinal photoreceptors during vertebrate eye development^45^. RAX2 is a member of RAX family, whose gene maps to chromosome 19p13.3 and is composed of three exons. The RAX2 gene (previously known as Q50-type retinal homeobox) has previously been related to cone-rod dystrophy^46^. Yang et al. found that the frameshift heterozygous mutation in RAX2 was related to mixed cone and rod dysfunction^47^. But the role of RAX2 in cancer is unclear. In the present study, we first characterized RAX2 as an oncogenic factor in glioblastoma cells. The expression level of RAX2 was upregulated in GT and increased as the pathological grade increased. Moreover, RAX2 knockdown inhibited the proliferation, migration and invasion, and promoted apoptosis in glioblastoma cells.

There are approximately more than one million copies of Alu elements in the human genome, which are mostly situated in lncRNAs and the 3'-UTR of mRNAs^48^. Alu elements have been reported to be associated with molecular mechanisms, such as silencing gene expression, gene conversion, and alternative splicing^49^. Recently, Alu elements were found to have functions in the transactivation of SMD. SMD is an mRNA decay process of mammalian cells which is mediated by STAU1 binding to a SBS within the 3'-UTR of target mRNAs^50^. STAU1 was first identified in drosophila which described to be ubiquitously expressed and involved in mRNA stability and translation in mammalian cells^51^. The SBSs can be formed by intramolecular base pairing within the target mRNA 3'-UTR or imperfect base pairing between an Alu element in the 3'-UTR of target mRNAs and a complementary Alu element in lncRNAs called ½-sbsRNAs^52^. The SBS-bound STAU1 recruits UPF1 and this event triggers rapid mRNAs degradation^53^. As a posttranscriptional regulatory pathway, SMD has modest effects on mRNA abundance and the diversity of cell-types. For instance, SMD has effects on the expression of target mRNAs, such as PAX3, ARF1, KLF2, and SERPINE1, which can regulate cellular homeostasis like myogenesis, adipogenesis and cutaneous wound healing in mammalian^24,54–56^. We found that an Alu element sequence in the 3'-UTR of RAX2 and another Alu sequence in BDNF-AS were found and predicted to have binding site with each other. With a variety of assays, we confirmed that BDNF-AS could bind to STAU1 and reduce the expression and stability of RAX2 mRNA via SMD, thereby further inhibited the malignant behaviors of glioblastoma cells. In a similar way, lncRNA-TINCR can degrade KLF2 mRNA expression through SMD, and promote cell cycle progression, and tumorigenicity subsequently in gastric cancer cells^16^.

DLG5 belongs to the MAGUK family, participates in epithelial cell polarity maintenance and cancer development^57^. Our results proved that DLG5 had a low expression level in glioma tissues and cells. Overexpression of DLG5 inhibited glioma cells malignant biological behaviors of glioblastoma cells. Similar results were reported in many human cancers, such as bladder cancer, prostate cancer, breast cancer, and hepatocellular carcinoma^19,20,58^. We also found that knockdown of RAX2 could increase DLG5 expression. Moreover, DLG5 promoter was predicted that it has RAX2-binding sites. Subsequently, we confirmed that RAX2 could bind to the promoter region of DLG5 and downregulate the promoter activities. The results indicated that DLG5 is involved in RAX2 regulation in glioblastoma cells.

The Hippo pathway is a evolutionarily conserved regulator of diverse biological processes including cell proliferation, tissue growth, and cancer development^59^. In mammalian, the core Hippo pathway consists of a kinase cascade and associated proteins including macrophage stimulating 1/2 kinase, Salvador homolog 1, and large tumor suppressor kinase 1/2 (LATS1/2)^60^. Yes-associated protein (YAP) is the major downstream effector of the Hippo pathway, which is identified as an oncogene. Activated YAP can translocate into the nucleus and bind to the TEAD transcription factor family to regulate cell proliferation, migration, epithelial–mesenchymal transition, and organ growth. Activation of the Hippo pathway results in the cytoplasmic retention and degradation of YAP mediated by LATS1/2 direct phosphorylation on YAP Ser127^61^. It had been demonstrated that inhibition of Hippo/YAP signaling promotes cell proliferation motility and invasion in glioma^62^, and the downstream effectors of Hippo pathway could be biomarkers of the prognosis of glioma patients^63^. Moreover, Liu et al. suggested that loss of DLG5 expression inhibited the Hippo pathway thus promoted breast cancer malignancy^26^. In this study, we first showed that overexpression of DLG5 activated the Hippo pathway by increasing the phosphorylation of YAP and suppressed the malignant behaviors of glioblastoma cells consequently.

In summary, we first found that PABPC1, BDNF-AS, and DLG5 were downregulated in human glioma tumors and cells, while RAX2 was upregulated conversely. Overexpression of PABPC1, BDNF-AS, and DLG5 along with depletion of RAX2 inhibited malignant biological behaviors of glioblastoma cells. PABPC1 could bind to and stabilize BDNF-AS, which promoted RAX2 mRNA degradation through SMD. Knockdown of RAX2 increased DLG5 expression thereby inhibited the malignant biological behaviors of glioblastoma cells by activating the Hippo pathway. Our findings first revealed that PABPC1/BDNF-AS/RAX2/DLG5 module plays an important role in glioblastoma cells, provided insight into potential therapeutic targets to treat glioma.

## Supplementary information

Figure S1

Figure S2

Figure S3

Additional File 1

Supplementary Figure Legends

## Data Availability

All data generated in this study are included within the article and its supplementary materials.
