# Supplementary figures and images for: PABPC1-induced stabilization of BDNF-AS inhibits malignant progression of glioblastoma cells through STAU1-mediated decay
Source: Cell Death Dis. 2020 Feb 3;11(2):81. doi: 10.1038/s41419-020-2267-9 (PMC6997171; doi:10.1038/s41419-020-2267-9)

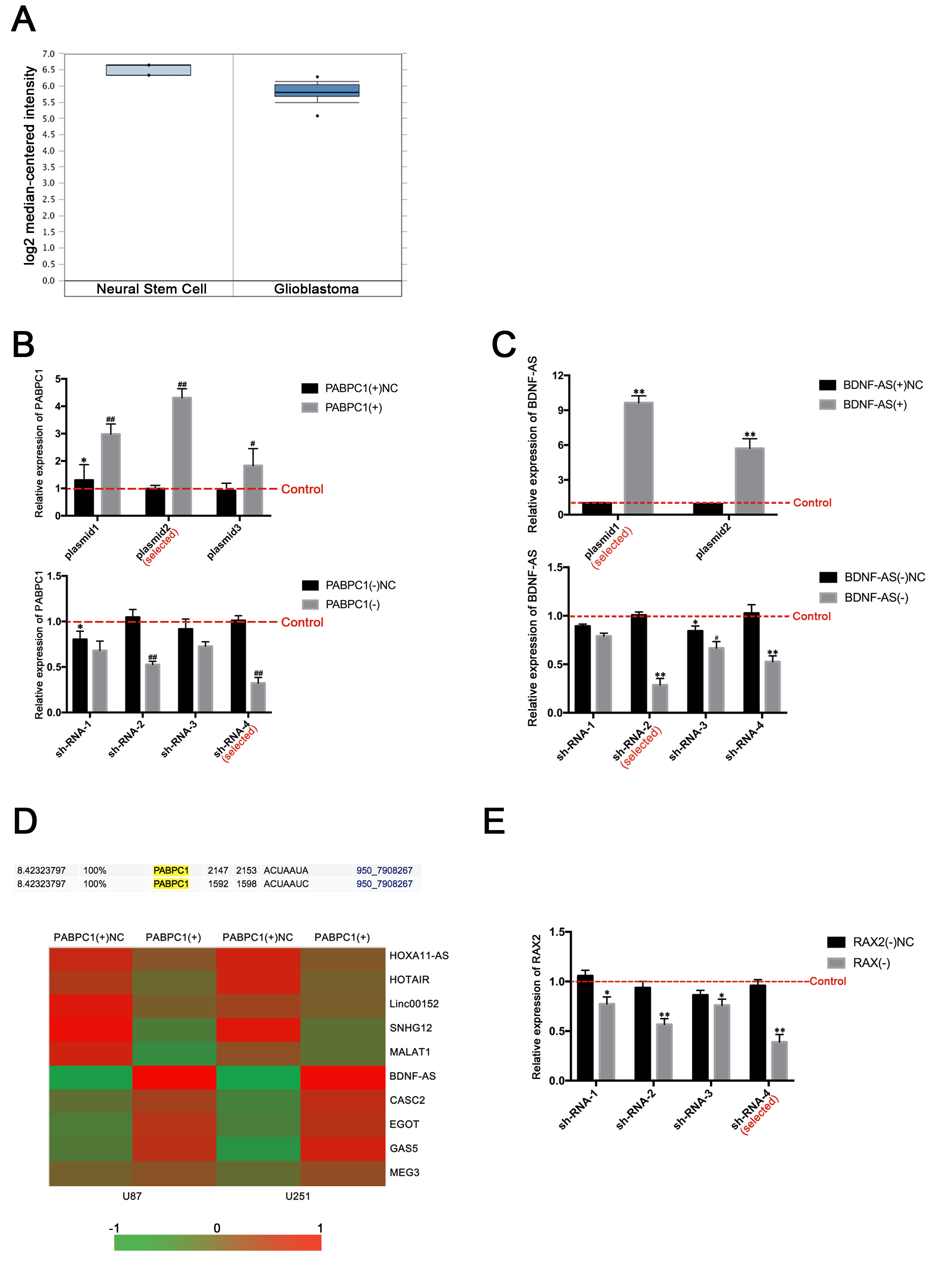

Supplement: Supplementary file 1 — Figure S1 [file 41419_2020_2267_MOESM1_ESM.tif]

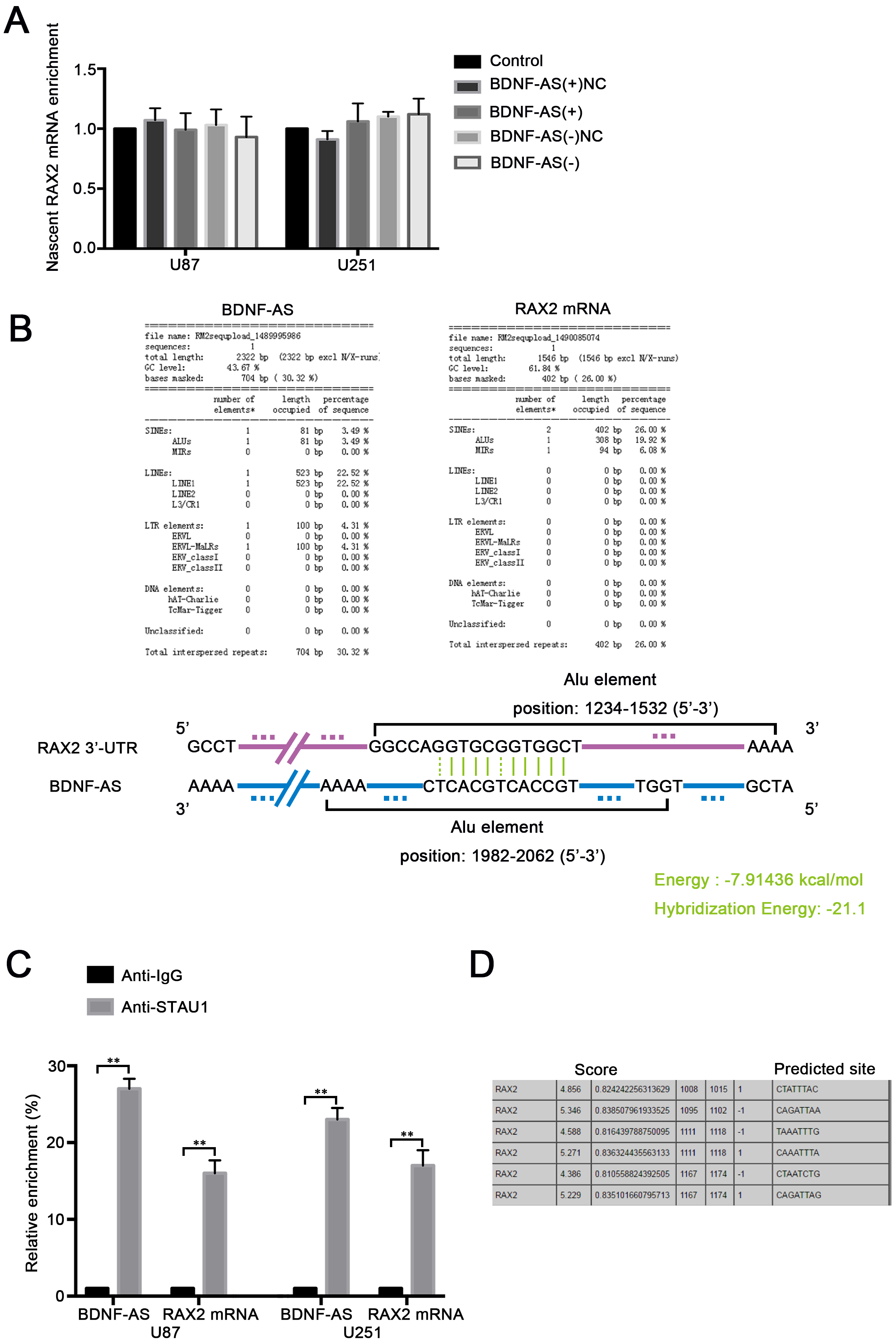

Supplement: Supplementary file 2 — Figure S2 [file 41419_2020_2267_MOESM2_ESM.tif]

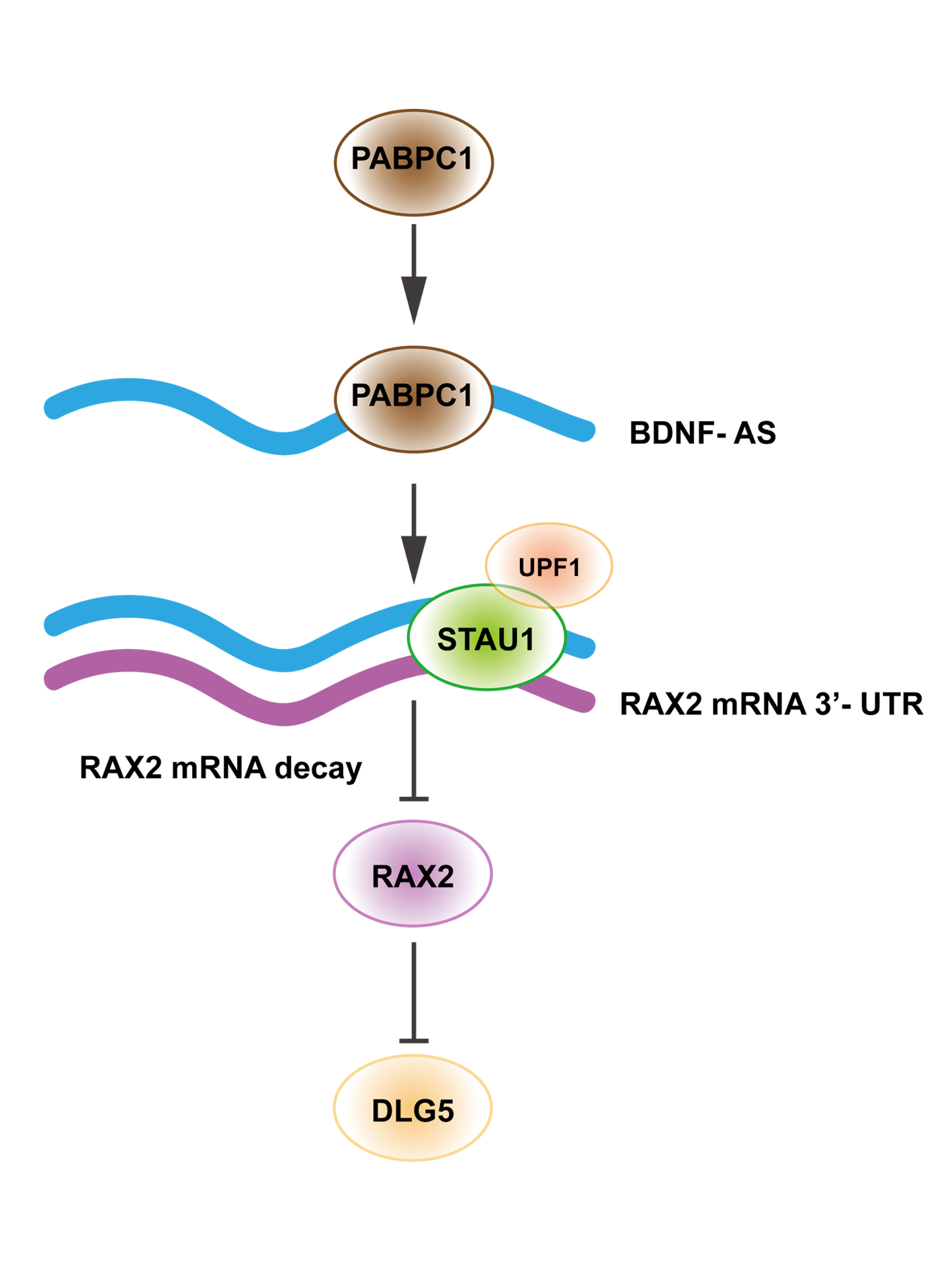

Supplement: Supplementary file 3 — Figure S3 [file 41419_2020_2267_MOESM3_ESM.tif]
