# Supplementary material for: PABPC1-induced stabilization of BDNF-AS inhibits malignant progression of glioblastoma cells through STAU1-mediated decay
Source: Cell Death Dis. 2020 Feb 3;11(2):81. doi: 10.1038/s41419-020-2267-9 (PMC6997171; doi:10.1038/s41419-020-2267-9)
Supplement: Supplementary file 4 — Additional File 1 [file 41419_2020_2267_MOESM4_ESM.docx]

| Gene Name | Sequence (5'->3') |
| --- | --- |
| PABPC1 | Forward primer: CACCGGTGTTCCAACTGTTT |
|  | Reverse primer: TGCTAGACCTGGCATTTGCT |
| BDNF-AS | Forward primer: TGCAAGGAGCCTGAGAACAA |
|  | Reverse primer: GCGCCAGCTCTAGTGACACA |
| RAX2 | Forward primer: CTCCCCAGATCACTCCCAGA |
|  | Reverse primer: TCTTGTGCTTACTGCCGTGT |
| DLG5 | Forward primer: CCCTACCACAGGCTGAATCC  Reverse primer: TGTCCACGTCATCCTTCAGC |
| GAPDH | Forward primer: CCCATCACCATCTTCCAGGAG |
|  | Reverse primer: GTTGTCATGGATGTCCTTGGC |

**RNA extraction and quantitative real-time PCR (qRT-PCR)**

Table 1. Primers used for qRT-PCR

One-Step SYBR PrimeScript RT-PCR cycling conditions were as follows: 5 minutes at 42°C, 10 seconds at 95°C, 40 cycles of 3 seconds at 95°C, and 30 seconds at 60°C.

The reverse transcription was set as follows: 30 minutes at 16°C, 30 minutes at 42°C, and 5 minutes at 85°C. PCR conditions were set as follows: 2 minutes at 50°C, 10 minutes at 95°C, 40 cycles of 15 seconds at 95°C and 1 minutes at 60°C. GAPDH was used as the endogenous control for PABPC1, BDNF-AS, RAX2 and DLG5. The relative expression levels were quantified as folder changes using the 2^-△△Ct^ methods.

**Chromatin immunoprecipitation (ChIP) assay**

Table 2. Primers used for ChIP experiments

| Binding site | | Sequence (5'->3') |  | Product size |
| --- | --- | --- | --- | --- |
| CTATTTAC | | Forward primer: GCTCTTCTCAAATGCTGGCG  Reverse primer: TGGGGAGGGTTTCCCCTAAA | | 158bp |
| CAAATTTA | | Forward primer: TTACCGCAGTTGAGGCGATT  Reverse primer: GGAGACAAATGGTGGGGAGG | | 135bp |
| CAGATTAG | | Forward primer: TTTAGGGGAAACCCTCCCCA 107bp  Reverse primer: CTGCTGGAGACCCCCTCGTA | | |
| Control |  | Forward primer: TTACCGCAGTTGAGGCGATT  Reverse primer: GGAGACAAATGGTGGGGAGG | | 199bp |

U87 and U251 cells were cross-linked with 1% formaldehyde and collected in lysis buffer. Immunoprecipitation was carried out overnight at 4℃ using antibody against RAX2 or normal IgG with gentle shaking respectively. 2% of lysates were removed before antibody supplemental as an input. Immune complexes were captured with Protein G Agarose Beads and eluted for 30 min at 65℃ with gentle vortexing. The DNA crosslinks were reversed by 5M NaCl and Proteinase K and purified. Immunoprecipitated DNA was amplified by PCR using their specific primers.
