# Supplementary material for: PABPC1-induced stabilization of BDNF-AS inhibits malignant progression of glioblastoma cells through STAU1-mediated decay
Source: Cell Death Dis. 2020 Feb 3;11(2):81. doi: 10.1038/s41419-020-2267-9 (PMC6997171; doi:10.1038/s41419-020-2267-9)
Supplement: Supplementary file 5 — Supplementary Figure Legends [file 41419_2020_2267_MOESM5_ESM.docx]

**Figure S1. A.** Oncomine database showed lower expression level in glioblastoma (n=22) than neural stem cell (n=3). **B.** The transfection efficiency of PABPC1 overexpression (upper portion) or knockdown (lower protion). Data are presented as the mean ± SD (n = 3, each group). **P* < 0.01 vs. control; ^##^*P* < 0.01, ^#^*P* < 0.05 vs. PABPC1(+)NC or PABPC1(-)NC group. **C.** The transfection efficiency of BDNF-AS overexpression (upper portion) or knockdown (lower protion). Data are presented as the mean ± SD (n = 3, each group). ***P* < 0.01 vs. BDNF-AS(+)NC or BDNF-AS(-)NC group, **P* < 0.05 vs. control group; ^#^*P* < 0.05 vs. BDNF-AS(-)NC group. **D.** Upper portion: The binding site between PABPC1 and BDNF-AS were predicted using RBPDB. Lower portion: Expression profile of lncRNAs in U87 and U251 regulated by PABPC1 over-expression. **E.** The transfection efficiency of RAX2 with four sh-RNA plasmids respectively. Data are presented as the mean ± SD (n = 3, each group). ***P* < 0.01, **P* < 0.05 vs. RAX2(-)NC group.

**Figure S2. A.** The nascent transcripts of RAX2 mRNA were measured after BDNF-AS overexpressed and knockdown (n=3). **B.** Upper portion: two Alu elements on BDNF-AS and 3’-UTR of RAX2 mRNA were predicted. Lower portion: Diagram of predicted Alu elements within RAX2 3’-UTR (purple) and BDNF-AS (blue) and the imperfect binding site (green). **C.** RIP experiments were used to detect the interaction of BDNF-AS or RAX2 mRNA with STAU1 in U87 and U251 (n = 3). ***P* < 0.01 vs. Anti-IgG group. **D.** The potential binding sites of DLG5 in the 1,500bp upstream promoter region of RAX2 were found by JASPAR.

**Figure S3. Graphic illustration of PABPC1/BDNF-AS/RAX2/DLG5 regulatory axis.**
